# Miniaturized Supercritical Fluid Chromatography Coupled with Ion Mobility Spectrometry: A Chip-Based Platform for Rapid Chiral and Complex Mixture Analysis

**DOI:** 10.1021/acs.analchem.5c00227

**Published:** 2025-04-05

**Authors:** Julius Schwieger, Klaus Welters, Christian Thoben, Alexander Nitschke, Stefan Zimmermann, Detlev Belder

**Affiliations:** † Institute of Analytical Chemistry, Leipzig University, Linnéstraße 3, Leipzig 04103, Germany; ‡ Institute of Electrical Engineering and Measurement Technology, Leibniz University Hannover, Appelstraße 9a, Hannover 30167, Germany

## Abstract

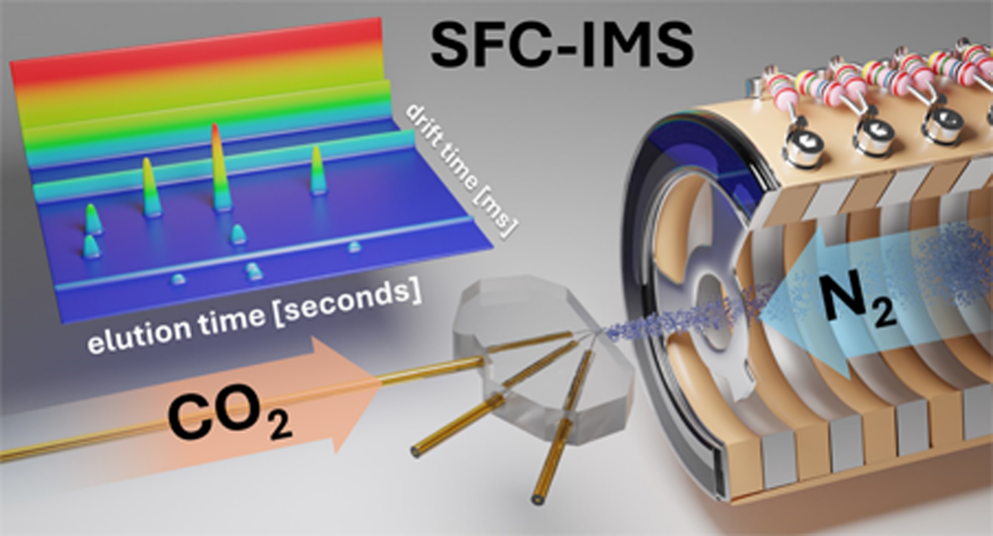

This study presents the first coupling of miniaturized chip-based supercritical fluid chromatography (SFC) with ion mobility spectrometry (IMS) enabling rapid two-dimensional analysis of moderately polar compounds. For the first time, ionization and analyte transfer at the SFC-IMS interface are achieved solely through eluent decompression in conjunction with a shifted electric IMS inlet potential. This straightforward approach significantly reduces instrumentation complexity and size, promoting system compactness and robustness. The integration of chip-based SFC with IMS enables high-speed separations of complex samples, drastically reducing analysis time while utilizing a detector capable of delivering structural information at a rapid acquisition rate and low cost. Evaluation of the SFC-IMS system as demonstrated through the chiral separation of Tröger’s base revealed exceptional repeatability and sensitivity. Short columns and high flow rates resulted in record-speed SFC-IMS analysis in just six seconds. The system was successfully used to analyze a complex mixture containing five isomers, including naloxone and 6-monoacetylmorphine, in just 30 s.

Advances in data science, combined with artificial intelligence and machine learning, have significantly accelerated the pace of modern data analysis. This progress has driven a growing demand for information, necessitating new technologies in analytical chemistry to generate measurement data at greater efficiency and speed.^1−4^ Consequently, numerous efforts have been directed to separation techniques, aiming to reduce analysis times to the range of seconds or even subseconds.^5−9^ However, in addition to speed, other aspects such as affordability, broad applicability, robustness, environmental friendliness and portability have become increasingly important. In light of these demands, supercritical fluid chromatography (SFC) is a highly promising separation technique.^10−12^ It utilizes supercritical carbon dioxide (scCO_2_), which has a high diffusion coefficient and low viscosity, enabling faster separations than conventional high-performance liquid chromatography (HPLC) while requiring lower pumping pressures.^13,14^ Moreover, the ability to implement both normal-phase methods and reverse-phase methods by adding a polar modifier makes SFC a versatile technique, offering promising potential for a wide range of applications in analytical chemistry.^15−17^

As far as SFC detection techniques are concerned, current research is primarily focused on coupling with mass spectrometry (MS).^18−22^ However, this approach is associated with considerable costs, both for instrumentation and maintenance, due to the need for high vacuum operating conditions. More economical alternatives such as ultraviolet (UV) or flame ionization detection (FID) are limited by their inability to provide comprehensive chemical information.^23,24^ In this context, ion mobility spectrometry (IMS) is a promising alternative. Although providing less specificity than MS, the technique is more cost-effective and robust while also being capable of providing a fast secondary separation dimension through characteristic ion mobilities, enhancing compound identification.^25−30^

Hill and coworkers made the first efforts to couple SFC to IMS in the late 1980s and 1990s.^31−34^ However, due to the numerous limitations of early SFC instrumentation, both SFC and its hyphenation capabilities gradually faded into the background.^24^ Today, SFC is experiencing a renaissance thanks to technological advances,^35^ but the exclusive coupling with IMS is still remarkably scarce.^36^ A possible reason for this is that commercial SFC has not yet reached the level of development seen in modern HPLC as well as the limited availability of commercial high-performance IMS in comparison to MS. However, significant progress regarding the miniaturization of SFC was reported recently, which opens up new possibilities for coupling with IMS.^10,37−39^

Inspired by this prospect and drawing on previous work with chip-based SFC platforms, we present a novel miniaturized SFC-IMS system. This approach significantly advances prior strategies by enabling the rapid and sensitive analysis of moderately polar substances while reducing instrumental effort, as analyte ionization and transfer are accomplished solely through eluent decompression toward the shifted electric potential of the IMS sample inlet.

## Experimental Section

### Chemicals and Materials

6-Acetylmorphine-D_3_/D_0_ (6-MAM, Cerilliant, reference material), naloxone (Nal, Cerilliant, reference material), boldine (Bol, analytical standard), 2-[3-(4,4-dimethyl-2,6-dioxocyclohexyl)propyl]isoindoline-1,3-dione (DPID, AldrichCPR R465704), 2-isopropylphenyl *N*-(4-(ethoxycarbonyl)phenyl)carbamate (IPPC, AldrichCPR S794295) and formic acid (FA) (LiChropur, 98–100% purity) were purchased from Merck KGaA (Darmstadt, Germany). The enantiomers (+)- and (−)-Tröger’s base were obtained from Honeywell Int. Inc. (Morristown, NJ, USA). HPLC-grade MeOH was purchased from VWR International LLC (Radnor, PA, USA). Pressurized CO_2_ (purity grade N45) for the SFC pump was purchased from Air Liquide S.A. (Paris, France). The chiral stationary phase IA-3 (fully porous, d_p_ = 3 μm) was obtained from Daicel (Osaka, Japan). The silica stationary phase Exsil Pure 120 Si (fully porous, 120 Å, d_p_ = 3 μm) was provided by Dr. Maisch HPLC GmbH (Ammerbuch-Entringen, Germany). Fluidic fittings and tubing were obtained from both VICI AG International (Schenkon, Switzerland) and IDEX Health and Science, LLC (Oak Harbor, WA, USA).

### SFC and IMS Instrumentation

Measurements were performed using the 1260 Infinity SFC System (Agilent Technologies Inc., Santa Clara, CA, USA). The integrated pump provided the eluent as variable mixtures of scCO_2_ and MeOH (0.1% FA) at a flow rate of 1.0 mL/min, while the temperature of the internal backpressure regulator (BPR) for flow splitting was set to 60 °C. The makeup liquid (MeOH) was supplied by a separate HPLC pump (LC-20AD, Shimadzu Corp., Kyoto, Japan) operating in constant flow mode, adjusted to maintain the required postcolumn pressure. No heating of the SFC capillary column was applied. Detailed information concerning the fabrication, valving, sample injection method, pressure regulation and microfluidic connection of the chip-based micro SFC platform is provided in previous work.^37^ To reduce condensation at the emitter tip, it was heated using a 3.2 W IR-LED flashlight equipped with a focusing lens (501BX–940 nm, Tianyida Electronic Tech. Co. Ltd., Shenzhen, PRC). Thermography measurements were performed with a FLIR ONE PRO thermal camera (Teledyne FLIR LLC, Wilsonville, OR, USA). The chip-based SFC platform was mounted on a 3D-printed holder made from a nonconductive copolyester filament (XT-CF20, ColorFabb B.V., Belfeld, Netherlands) and positioned relative to the IMS with a manual translational stage (T12XYZ, Thorlabs GmbH, Bergkirchen, Germany).

The chip-based SFC platform was coupled with a custom-built drift tube IMS with high resolving power, featuring a shifted electric inlet potential. The device was developed by the Zimmermann’s group based on previous designs with a shifted potential, incorporating an additional high voltage power supply to control the inlet potential independent of the drift potential. An overview of the instrument and operating parameters is given in Table 1. The device was controlled via a self-built software, implemented using LABView 2018 (National Instruments, Austin, TX, USA). If not stated otherwise, IMS raw data was filtered using a 10 kHz low-pass filter and subsequently processed using MATLAB (version R2022a, Mathworks, Natick, MA, USA) and/or OriginPro 2019 (Origin Lab Corporation, Northampton, MA, USA). The recorded drift time spectra were converted into inverse reduced ion mobility values using the following equation:
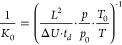
1

**Table 1:** IMS Instrument and Operating Parameters

electric inlet potential	–6.5 kV	measurement time	5.5–15.5 ms
drift voltage	9.6 kV	drift gas flow rate N_2_	250 mL/min
desolvation length	81 mm	pressure	1001–1004 hPa
drift tube length	77 mm	temperature	297–304 K
injection time	20 μs		

The inverse reduced ion mobility (1/*K*_0_) is calculated using drift tube length *L*, drift voltage Δ*U*, drift time *t*_d_, pressure *p* and temperature *T*. The pressure and temperature for each measurement were obtained from the IMS-integrated sensors. Standard temperature *T*_0_ is defined as 273.15 K, standard pressure *p*_0_ is defined as 1013.25 hPa.

## Results and Discussion

Based on a recently developed micro SFC-MS system^37^ and encouraged by the rapid development of ever faster and higher resolving power IMS devices, this work introduces the first coupling of chip-based SFC and IMS. It aims at achieving high-speed SFC-separations while providing a robust, low-cost, fast and comprehensive detection technique through IMS.

The chip-based SFC platform used in this study is based on a recently developed setup for SFC-MS.^37^ It contains two SLE-fabricated chip modules interconnected by a capillary column. The key features are the pinhole emitter and the microfluidic dilution-free postcolumn BPR. In conjunction, they allow for the decompression of the CO_2_-based eluent through a micrometer-sized emitter channel while preserving pressure flexibility and sample integrity.

**Figure 1:**
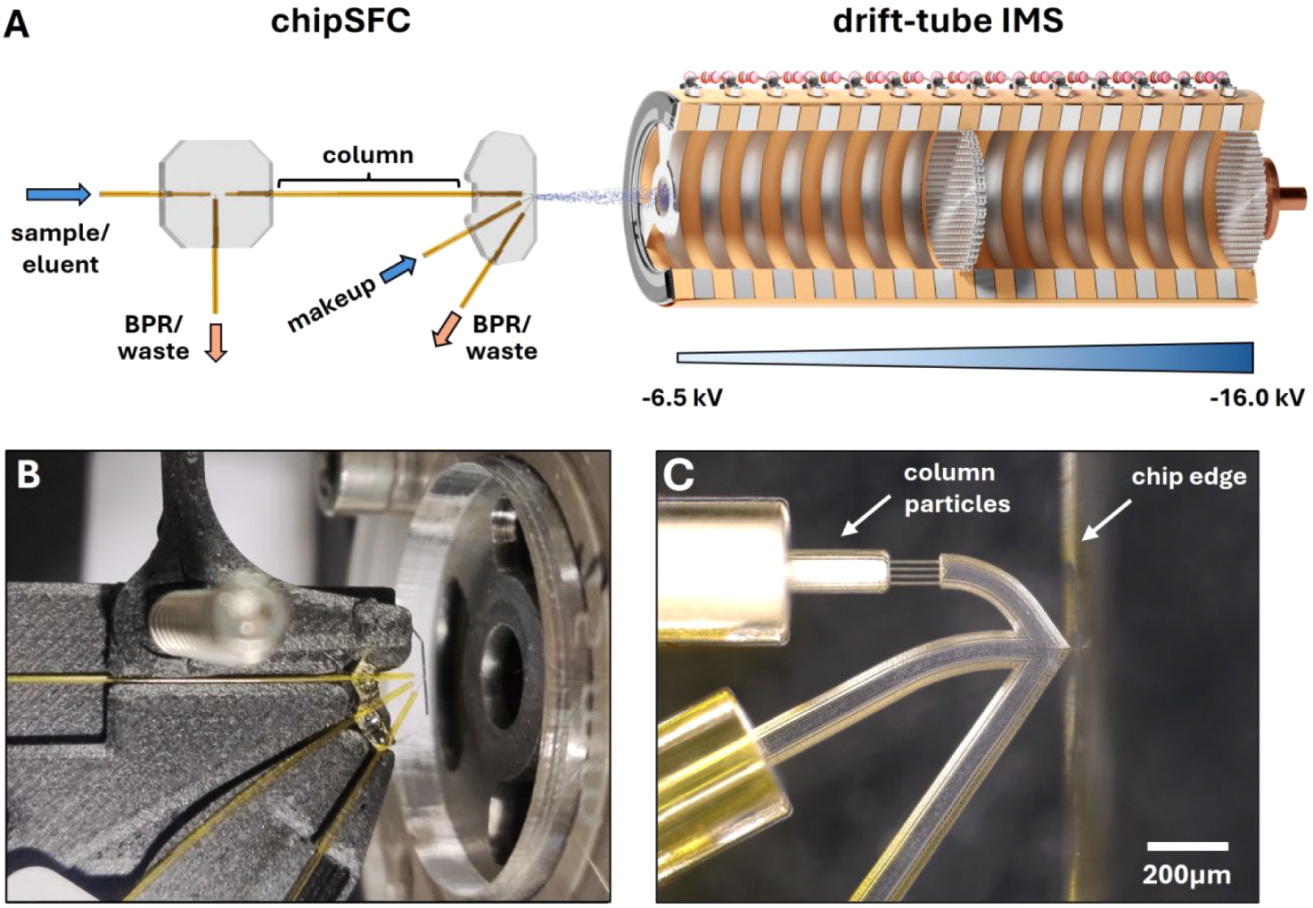
(A) Schematic representation of the SFC-IMS coupling. The chipSFC consist of a tee-junction chip (left) and an emitter chip (right). An exemplary drift tube IMS with tristate ion shutter is depicted in a sectional view. The white and blue bar indicates the electric potential gradient. (B) Placement of the SFC emitter chip in front of the IMS sample inlet. The system is fixed to a 3D printed holder system. (C) Microscopy image of the channel design on the emitter chip (without fluid). Column particles are retained by the comb-shaped μ-frit structure. The μm-sized emitter structure is located at the closest distance between channel and chip edge. The two empty channels serve as makeup inlet (second from top) and outlet (BPR). The makeup flow allows for the adjustment of the postcolumn pressure during measurements.

The SFC and drift tube IMS hyphenation was performed straightforwardly by positioning the chip-based SFC platform in coaxial alignment to the IMS sample inlet (Figure 1). This approach presents two significant challenges compared to existing SFC and IMS hyphenations. First, while the decreased pressure in the inlet capillary in SFC-MS provides unidirectional flow, the drift tube IMS employs a counter-flowing drift gas. The opposing flow directions of the expanding eluent and the drift gas threaten spray stability and the unhindered entry of analytes into the drift tube. Second, the instrumental setup typically employed for analyte transfer in ESI-IMS is not applicable in this context since the nonconductive nature of the eluent does not allow for the implementation of conventional contacting strategies.^40^ As a result, initial experiments using standard IMS devices with grounded inlets were unsuccessful, even when a potential was applied at both eluent and makeup flow through stainless steel unions. Neither analytes nor eluent could be detected by the IMS.

However, employing a high IMS inlet potential of –6.5 kV using a custom-built IMS device proved successful. Since the signal intensity was not influenced by electrical contacting of the eluent or makeup, it was omitted. The precise reason for the observed phenomenon is unclear, as the underlying ionization mechanism is still poorly understood. One possibility is that the high inlet potential and therefore the high electric field may only be necessary to attract the analyte ions formed during eluent decompression against the drift gas stream. This is supported by the observation that minimizing the transfer distance by placing the emitter close to the IMS inlet (3–6 mm), enhanced the IMS signal response. Since the potential of the drift region is increased by the inlet potential, thus reaching potentials of –16 kV against ground at the detector, voltages above –6.5 kV were not applied due to instrumental restraints.

Additionally, it must be noted that the IMS inlet was not heated initially, contrary to the hyphenation with MS.^37^ Therefore, the eluent decompression resulted in a strong cooling of the region adjacent to the emitter structure, causing condensation of the modifier component on the chip and, in some cases, even dry ice formation. To mitigate these effects that compromise spray stability, an IR lamp was focused on the emitter, enabling effective contactless heating of the emitter up to ca. 60 °C (Figure S1).

### Evaluation of the Coupling Using Model Compounds

**Figure 2:**
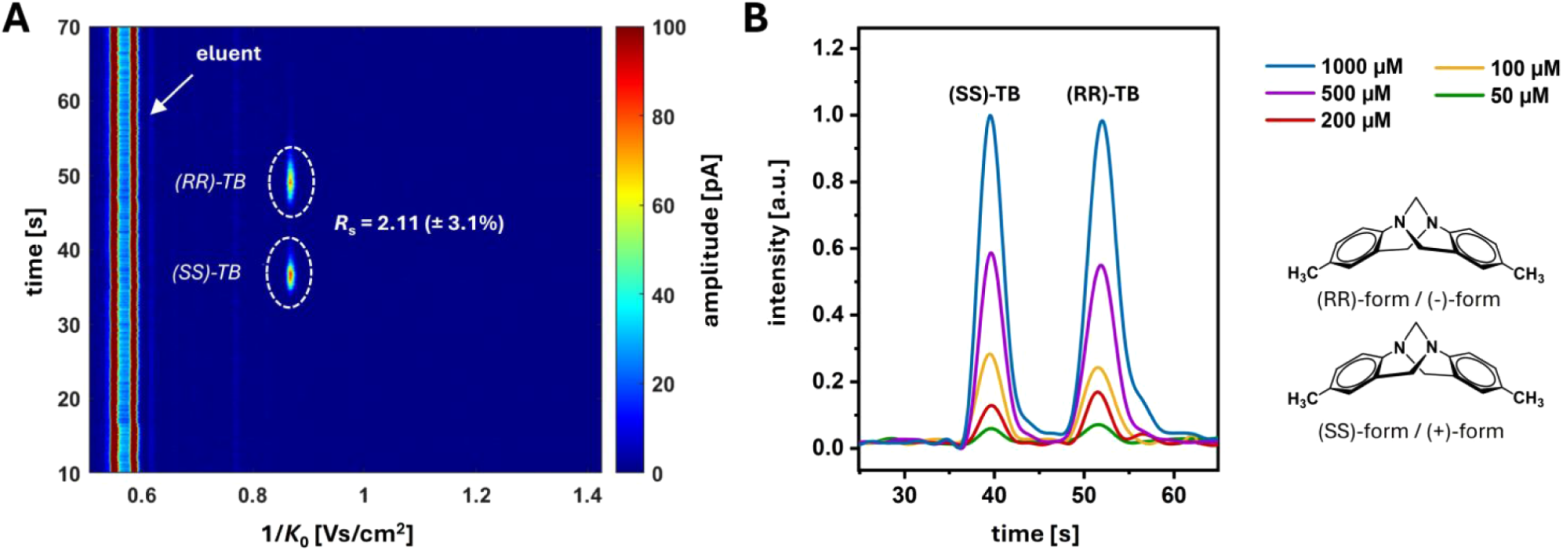
(A) 2D plot of the separation of Tröger’s base, signal intensity plotted against (elution) time and inverse reduced ion mobility 1/*K***_0_**. Sample: 4 nL of 1 mM Tröger’s base (racemate) in MeOH; eluent: 90/10 (v/v) CO_2_:MeOH (0.1% FA); column: 8.7 cm, IA-3 (*d*_p_ = 3 μm), *T* = 25 °C, *p*_precolumn_ = 143 bar, *p*_postcolumn_ = 95 bar; MeOH makeup flow: 10 μL/min; shifted IMS inlet voltage: –6.5 kV; acquisition rate: 10 Hz. Calculated *K*_0_ value: 1.223 cm^2^/(V s). The RSD of the *R*_s_ value was calculated based on three measurements. (B) Separation of Tröger’s base using different sample concentrations (50–1000 μM). Conditions equal to A, except for the following: eluent: 85/15 (v/v) CO_2_:MeOH (0.1% FA), *p*_precolumn_ = 143 bar, *p*_postcolumn_ = 93 bar; Chromatograms were processed with a 15 point Lowess filter.

To evaluate the performance of the SFC-IMS system, a chiral separation of a racemic mixture of Tröger’s base (2,8-Dimethyl-6*H*,12*H*-5,11-methanodibenzo[*b*,*f*][1,5]diazocine) was chosen. Since enantiomers exhibit identical *K*_0_ values, they cannot be analyzed by IMS alone.^41^ Baseline separation of the (RR)- and (SS)-enantiomer was achieved in less than 60 s using an 8.7 cm long IA-3 capillary column with an inner diameter (ID) of 100 μm and 10% modifier (MeOH, 0.1% FA) in the eluent. A representative measurement is shown in Figure 2A. The elution order was determined using standard solutions of the individual enantiomers. Three consecutive measurements yielded a chromatographic resolution of *R*_s_ = 2.11 (±3.1%). The relative standard deviation (RSD) values of the chromatographic performance parameters, e.g., elution time (0.3%) and peak area (3.7–6.3%) were low. A comprehensive overview of the chromatographic performance parameters is given in Table 2. Throughout the measurement series, the intensity and position of the eluent signals remained constant, as shown in Figure 2A, indicating excellent spray stability during the analysis. To evaluate the influence of the composition of the eluent on the separation, the process was repeated with varying amounts of modifier (2.5–22.5%). The measurements (Figure S2) showed that an increased eluent polarity accelerated the separation only slightly with the lowest elution times at 17.5–20.0%, while the chromatographic resolution linearly reduced from 2.48 (±0.7%) to 1.59 (±0.9%). At the same time, both signal intensity and peak area increased by up to 106% and 121%, respectively, indicating a significant influence of the modifier on ionization efficiency. To determine the sensitivity and thus the limit of detection (LOD) for separating Tröger’s base at a given modifier fraction of 15%, analyte concentration was successively reduced, starting from 1 mM. The LOD value (S/N ≈ 3) was found to be approximately 50 μM of sample concentration. Considering the findings of previous work,^38^ it can be assumed that only 7.2% of the 4 nL sample (equivalent to 14.4 picomoles) was injected onto the column, thus underscoring the excellent sensitivity of the SFC-IMS system. For the analyzed concentration range of 50–1000 μM a linear fit of the IMS signal response versus sample concentration yielded an R^2^ value of 0.998 (Figure S3).

**Table 2:** Chromatographic Performance Parameters of Tröger’s Base Separation (Figure 1A)

Peak	Elution time [s]^a^	Full width at half-maximum [s]^a^	Peak area [a.u.]^a^
(SS)-TB	36.7 (0.3%)	2.9 (2.7%)	1.07 (3.7%)
(RR)-TB	49.3 (0.3%)	4.2 (3.4%)	1.28 (6.3%)

a results presented as mean (RSD in %) of three measurements.

**Figure 3:**
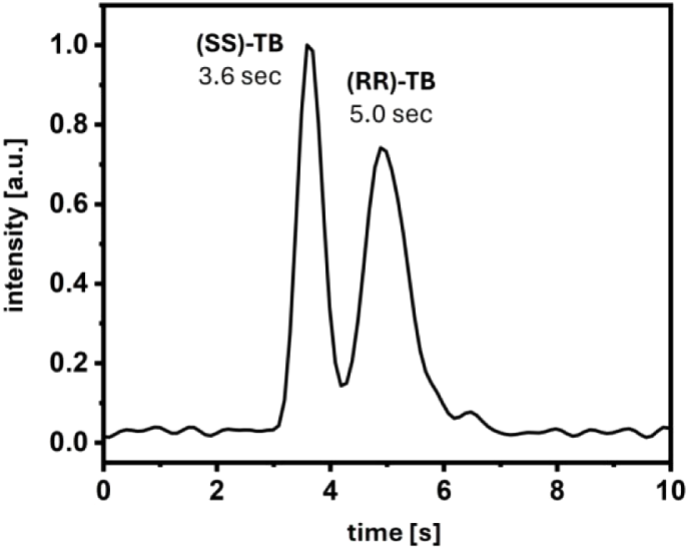
Fast separation of Tröger’s base. Sample: 4 nL of 1 mM Tröger’s base (racemate) dissolved in MeOH; eluent: 90/10 (v/v) CO_2_:MeOH (0.1% FA); column: 4.0 cm, IA-3 (*d*_p_ = 3 μm), *T* = 25 °C, *p*_precolumn_ = 190 bar, *p*_postcolumn_ = 101 bar; MeOH makeup flow: 15 μL/min; IMS inlet voltage: –6.5 kV; acquisition rate: 10 Hz, chromatogram was processed with a two point FFT filter.

Subsequently, the evaluation of the system was further extended to determine the applicability of SFC-MS to high-throughput methods. This area presents significant interest, as IMS technology currently supports acquisition rates up to 2000 Hz,^42^ enabling signal recording within the subsecond time scale.^7^ These high acquisition rates are compatible with the ultrafast separation capabilities of SFC, underscoring the potential of SFC-IMS for rapid screening analyses. Investigations were conducted to accelerate the chiral separation of Tröger’s base by gradually increasing the pressure drop along the column from 65 to 108 bar. The results are presented in Figure S4. While this approach enhanced chromatographic resolution, it also reduced the analysis time by approximately 45%, from 42 to 23 s. Since the fastest analysis required an inlet pressure of 222 bar, thus reaching the applicable pressure limit of both the connected BPRs and the fluidic connections, a further pressure increase could not be realized with the current instrumental setup. Therefore, to further accelerate the separation, the chromatographic column was shortened from 8.7 to 4.0 cm, reducing backpressure and consequently increasing the eluent flow rate. The obtained measurement is presented in Figure 3. At an analysis time of six seconds (including the sample transport from the external valve to the column head), the two enantiomers were near baseline separated, reaching an *R*_s_ value of 1.20. This result demonstrates the suitability of the SFC-IMS system for high-throughput applications.

### Application: Drug Analysis of Isomeric Mixtures

The SFC-IMS system was further tested in drug analysis using a complex mixture of structural isomers. Such mixtures cannot be analyzed by single-stage MS and require more elaborate MS instruments capable of fragmentation experiments. Therefore, IMS is a promising, low-cost and portable alternative with a separation mechanism that is not based on the mass-to-charge ratio but on mobility and thus size, shape and charge. However, since isomers may still exhibit similar ion mobilities (and thus drift times) and simultaneous analysis of multiple substances can be impeded by adduct formation and ion suppression effects depending on the ionization mechanism, incorporating a second separation dimension via SFC is essential.

**Figure 4:**
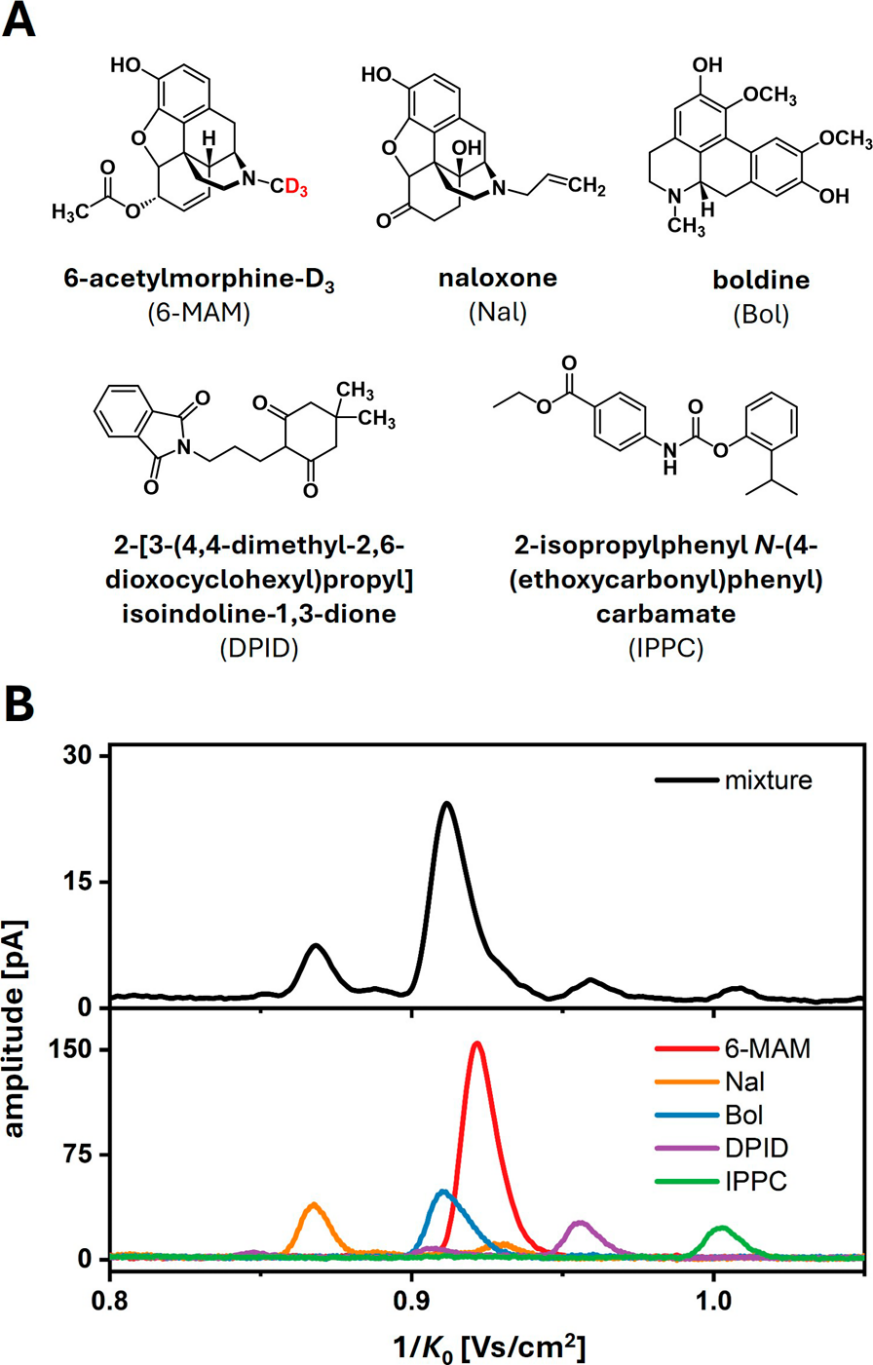
(A) Structural formulas of the mixture components. (B) Inverse reduced ion mobility plots of direct infusion ESI-IMS measurements using the mixture (top) and the individual components (bottom). Sample: 100 μM solution per component (80/20 (v/v) MeOH:water), 1 μL/min flow rate, –5.5 kV ESI voltage. The instrumental ESI-IMS setup is presented in Figure S5.

The mixture used for the experimental trial consisted of five compounds: 6-acetylmorphine-D_3_ (6-MAM), naloxone (Nal), boldine (Bol), 2-[3-(4,4-dimethyl-2,6-dioxocyclohexyl)propyl]isoindoline-1,3-dione (DPID) and 2-isopropylphenyl *N*-(4-(ethoxycarbonyl)phenyl)carbamate (IPPC). 6-Acetylmorphine is a potent drug and as the primary metabolite of heroin employed in screenings for drug abuse. Due to legal restrictions, it was purchased as a deuterated solution. The difference in ion mobility between the presented measurements and those of the non-deuterated compound, as measured by separate direct infusion ESI-IMS experiments (Figure S5), was found negligible.^43^ DPID and IPPC are synthetic building block substances and were included to represent the high sample complexity typically found in drug screenings.^44^ The molecular mass of all substances (if non-deuterated) is 327.37 g/mol, with structural formulas depicted in Figure 4A.

Prior to SFC-IMS experiments, all compounds were individually measured using direct infusion ESI-IMS to determine characteristic drift times and *K*_0_ values. To evaluate the necessity of a front-end separation technique, a sample mixture was analyzed as well. Figures 4B and S6 present a comprehensive overview of the results. In the ESI-IMS measurements of the individual substances, strong monomer signals were observed for each compound at lower inverse reduced ion mobilities (1/*K*_0_: 0.80–1.05 Vs/cm^2^). Weaker signals, presumably corresponding to multimer clusters (1/*K*_0_ > 1.2 Vs/cm^2^), were visible for some components (Figure S6). Since these multimer signals typically arise at high sample concentrations and exhibit varying intensities in the presence of other compounds, also resulting in the formation of mixed multimers, they could not be used for clear compound identification. Furthermore, due to the partial overlap of monomer signals in the sample mixture, they also proved insufficient for identification through ESI-IMS, despite the high resolving power of the IMS device of *R*_p_∼80. Additionally, the presence of multiple substances led to a significant decrease in individual signal intensities due to charge competition, thereby reducing overall sensitivity. As a result, incorporating SFC as a fast additional separation dimension is necessary to achieve adequate analysis in this experimental context.

**Figure 5:**
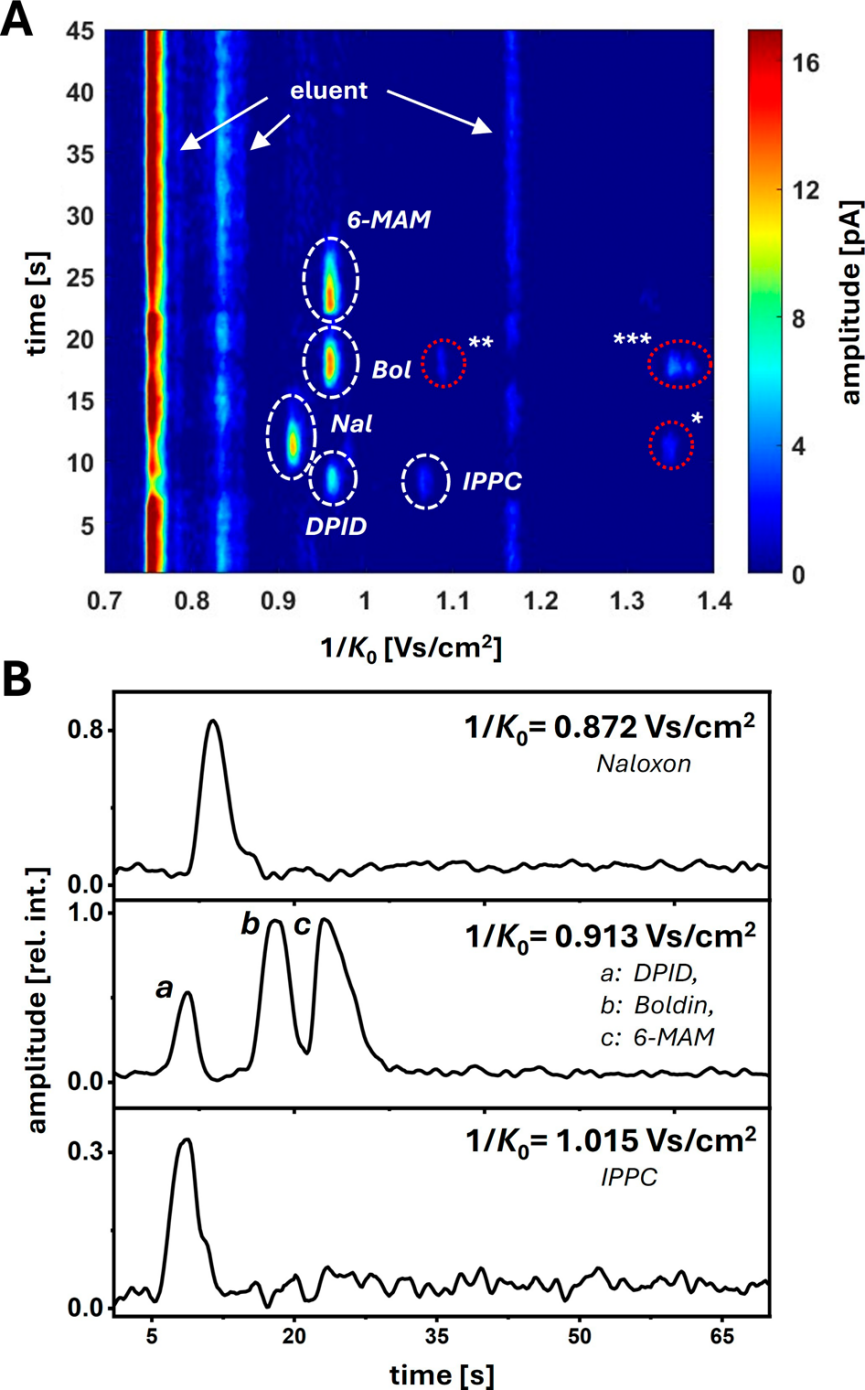
(A) 2D plot of the separation of the isomers, signal intensity plotted against elution time and inverse reduced mobility. The signal marked with * corresponds to a visible multimer of naloxone, the signals marked with ^**^ and ^***^ correspond to multimers of boldine. Sample: 4 nL of 1 mM each 6-acetylmorphine-D_3_ (6-MAM), naloxone (Nal), boldine (Bol), 2-[3-(4,4-dimethyl-2,6-dioxocyclohexyl)propyl]isoindoline-1,3-dione (DPID) and 2-isopropylphenyl *N*-(4-(ethoxycarbonyl)phenyl)carbamate (IPPC) in MeOH; eluent: 40/60 (v/v) CO_2_:MeOH (0.1% FA); column: 3.7 cm, Exsil Pure 120 Si (fully porous, *d*_p_ = 3 μm), *T* = 25 °C, *p*_precolumn_ = 185 bar, *p*_postcolumn_ = 107 bar; makeup flow: 3 μL/min; IMS inlet voltage: –6.5 kV; acquisition rate: 10 Hz. A 5 kHz lowpass filter was employed for data processing. (B) Extracted chromatograms according to the specified 1/*K*_0_ values. Chromatograms were processed with a three-point median filter.

Following the direct infusion ESI-IMS experiments, separation of the components via the chip-based SFC-IMS was performed in less than 30 s, using bare silica as the stationary phase (3 μm), a short column (37 mm) and a high modifier percentage (60%). The results are presented in Figure 5. The signal assignment was performed based on *K*_0_ values obtained by ESI-IMS and confirmed by SFC-IMS measurements of the individual components. It must be noted that while SFC is best suited for nonpolar substances, the separation conditions employed in this study (less than 50% CO_2_) are commonly classified as enhanced-fluidity liquid chromatography (EFLC). Therefore, depending on the eluent composition, the presented system may not only be described as an SFC-IMS hyphenation but is also fully compatible with the use of EFLC-IMS, allowing for an increased range of accessible analyte polarity.

In the case of the isomer mixture, this allowed for the separation of all five components. Except for boldine and 6-acetylmorphine-D_3_ all compounds were baseline separated by elution time, drift time, or a combination of both. Surprisingly, the reduced ion mobility of DPID measured in SFC-IMS differed from previous values found in ESI-IMS measurements. This might be due to differences in the ionization or desolvation mechanism. As a result, DPID exhibited a reduced ion mobility similar to boldine and 6-acetylmorphine-D_3_. However, chromatographic separation of these compounds was achieved with corresponding *R*_S_ values of 2.19 and 1.01, respectively (Figure 5B). Coelution was only observed for DPID and IPPC, causing decreased signal intensities due to charge competition. However, the significant difference in reduced ion mobility afforded clear separation. Despite moderate signal intensities, multimer signals were observed for some substances during measurements. This might be associated with temperature effects relating to CO_2_ decompression at the emitter or the eluent composition.

## Conclusion

Herein, we present a straightforward chip-based SFC-IMS system with atmospheric pressure ionization to analyze moderately polar substances. The absence of additional instrumentation at the interface allowed for simplified assembly while providing excellent repeatability of the chromatographic performance parameters. The combination of miniaturized SFC and a custom-built IMS with shifted electric inlet potential allowed for a compact and low-cost alternative to SFC-MS, providing structural information on the analytes in the additional separation dimension. A rapid chiral analysis of Tröger’s base in only 23 s demonstrated the system’s applicability for high-throughput methods. Near-baseline separation was even achieved within 6 s, establishing these separations to the best of our knowledge as the fastest for SFC-IMS reported to date. The novel coupling system was tested for achiral analysis of drug-containing isomeric mixtures, revealing fast separation of a complex sample in less than 30 s. The presented results reflect the promising potential of SFC-IMS and mark a vital step in developing rapid and portable SFC-based analytical devices.

## Supporting Information Available

Detailed information about the IR heating of the emitter chip, the influence of the modifier fraction and pressure on the chromatographic separation of Tröger’s base, the concentration dependence of the IMS signal response, and a description of the ESI-IMS setup, including corresponding measurements of the structural isomers (PDF)
