# Supplementary material for: Miniaturized Supercritical Fluid Chromatography Coupled with Ion Mobility Spectrometry: A Chip-Based Platform for Rapid Chiral and Complex Mixture Analysis
Source: Anal Chem. 2025 Apr 5;97(14):7954–60. doi: 10.1021/acs.analchem.5c00227 (PMC12004342; doi:10.1021/acs.analchem.5c00227)
Supplement: Supplementary file 1 — ac5c00227_si_001.pdf [file ac5c00227_si_001.pdf]

SUPPORTING INFORMATION

Miniaturized Supercritical Fluid Chromatography Coupled with Ion Mobility Spectrometry: A Chip-Based platform for Rapid Chiral and Complex Mixture Analysis

Julius Schwieger[a], Klaus Welters[a], Christian Thoben[b], Alexander Nitschke[b], Stefan Zimmermann[b], Detlev Belder[a]*

[a]*Institute of Analytical Chemistry, Leipzig University, Linnéstraße 3, 04103 Leipzig, Germany*

[b]*Leibniz University Hannover, Institute of Electrical Engineering and Measurement Technology, Department of Sensors and Measurement Technology, Appelstraße 9A, 30167 Hannover, Germany*

Corresponding author:

*E-mail Detlev Belder: belder@uni-leipzig.de

Table of contents

Figure S1	–	Temperature profile of the emitter chip during heating with an IR lamp	page – S2
Figure S2	–	Influence of the modifier fraction on the enantiomeric separation of Tröger base	page – S3
Figure S3	–	Dependence of the signal response of Tröger's base on sample concentration	page – S4
Figure S4	–	Enantiomeric separation of Trögers base at different pressure drops along the column	page – S5
Figure S5	–	ESI-IMS measurements of 6-MAM D_3 and 6-MAM D_0	page – S6
Figure S6	–	Inversed reduced mobility plot of the individual mixture components and the mixture	page – S7
Figure S7	–	Drift time spectra of SFC-IMS and ESI-IMS solvent	page – S8

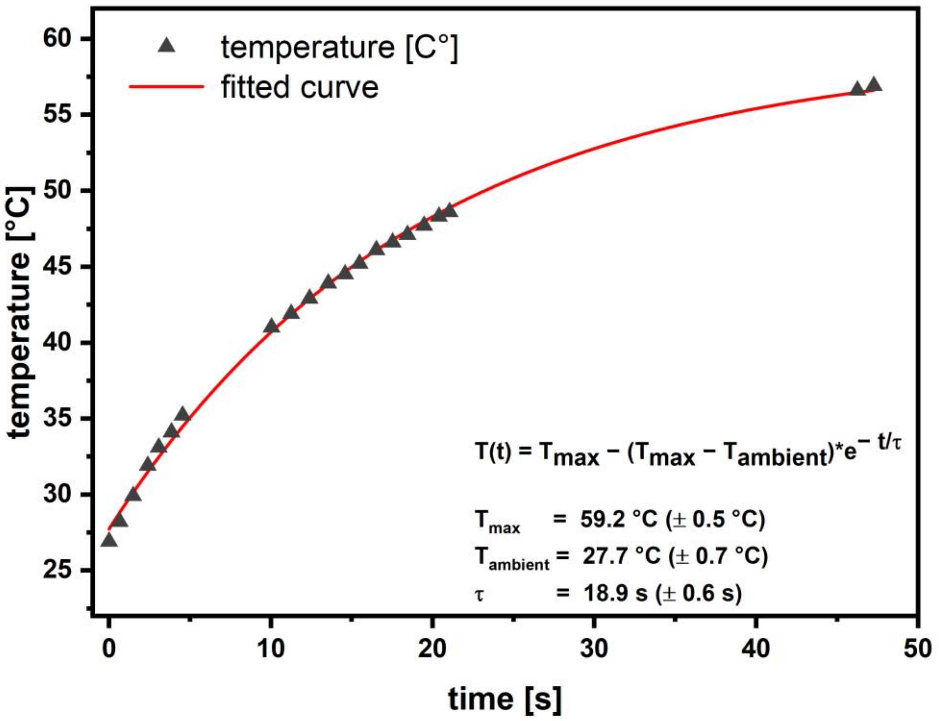

Figure S1 – Temperature profile of the emitter chip during heating with an IR lamp. The temperature curve was plotted using limited growth function. During measurements the IR-LED flashlight (3.2 W, 501BX-940nm, Tianyida Electronic Tech. Co. Ltd., Shenzhen, PRC) was positioned approximately 20 cm above the chip-based SFC platform. Focusing of the lamp on the chip was verified using a mobile phone camera to make the IR radiation visible. Measuring of the surface temperature was conducted using a FLIR ONE PRO thermal camera (Teledyne FLIR LLC, Wilsonville, OR, USA). A maximum temperature of 59 °C was reached after one minute of irradiation.

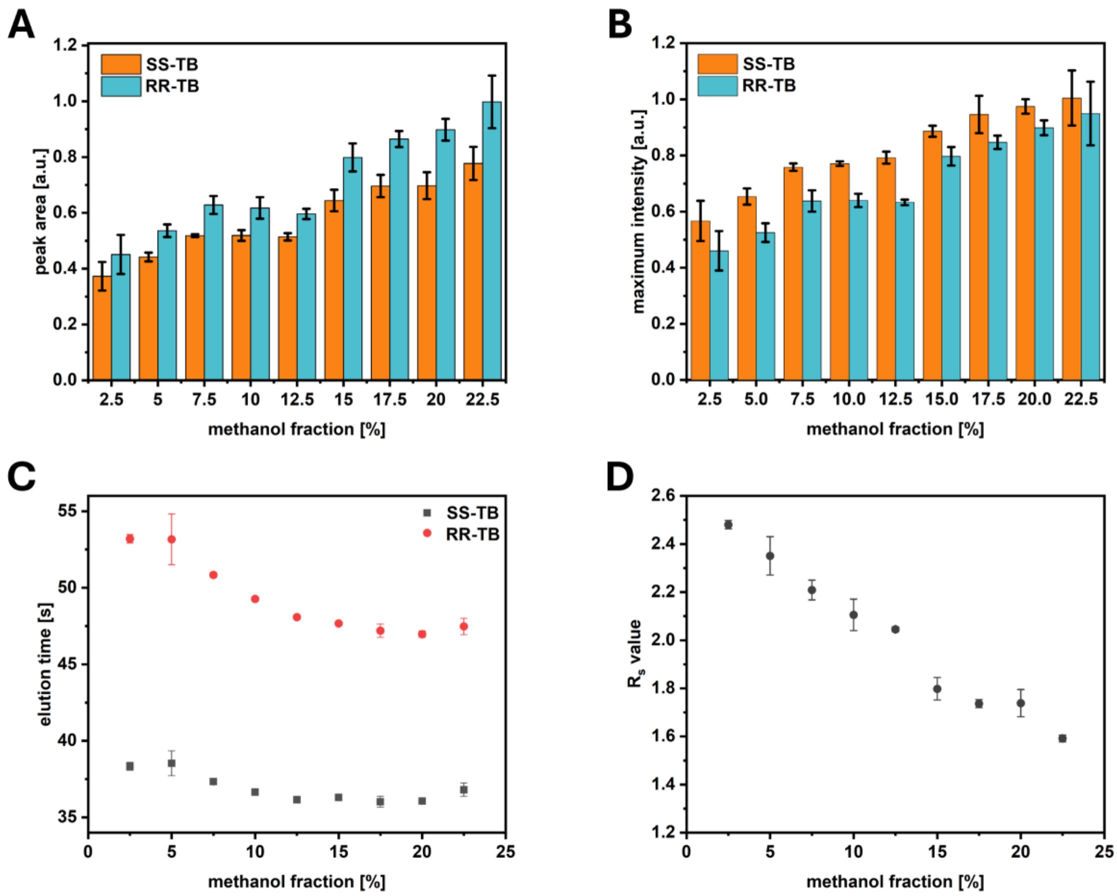

Figure S2 – Influence of the modifier fraction on the enantiomeric separation of Tröger base. The modifier fraction was increased from 2.5% to 22.5 %. Its influence was evaluated on (**A**) the peak area, (**B**) the intensity, (**C**) the elution time and (**D**) the chromatographic resolution (R_s) value. Sample: 4 nL of 1 mM Tröger's base (racemate) in MeOH; eluent: CO_2 and MeOH (0.1% FA) in varying ratios; column: 8.7 cm, IA-3, T = 25 °C, $p_{precolumn}$ = 143 bar, $p_{postcolumn}$ = 93-95 bar; MeOH makeup flow: 10 µL/min; shifted IMS inlet voltage: −6.5 kV, acquisition rate: 10 Hz.

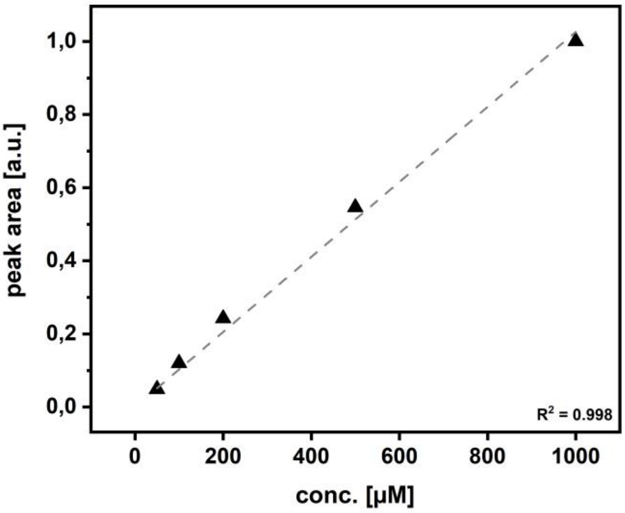

Figure S3 – Dependence of the signal response of Tröger's base (RR-form) on sample concentration. Sample: 4 nL of 1 mM Tröger's base (racemate) in MeOH; eluent: 85:15 v/v CO_2:MeOH (0.1% FA); column: 8.7 cm, IA-3, T = 25 °C, $p_{precolumn}$ = 143 bar, $p_{postcolumn}$ = 93 bar; MeOH makeup flow: 10 µL/min; shifted IMS inlet voltage: −6.5 kV, acquisition rate: 10 Hz.

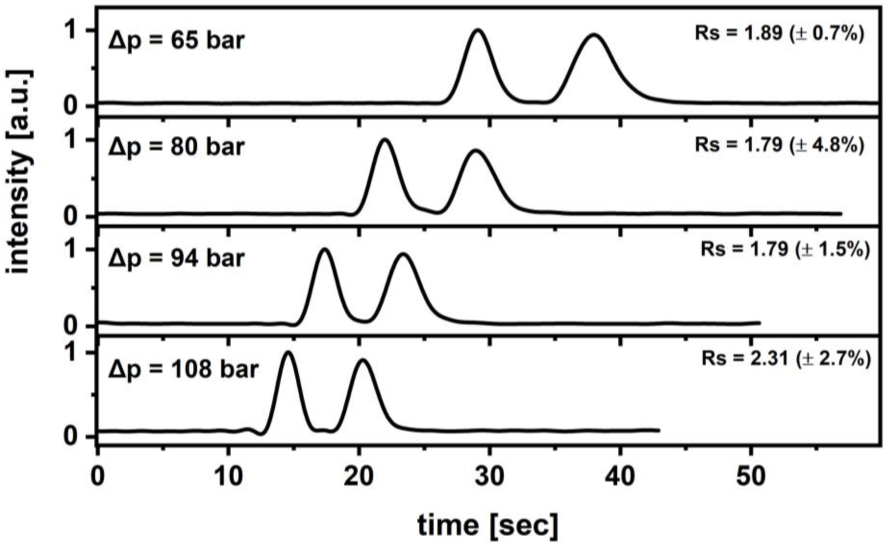

Figure S4 – Enantiomeric separation of Trögers base at different pressure drops along the column. R_s values were calculated based on three consecutive measurements. Sample: 4 nL of 1 mM Tröger's base (racemate) in MeOH; eluent: 85:15 v/v CO_2:MeOH (0.1% FA); column: 8.7 cm, IA-3, T = 25 °C, pressure drop: 163-98 bar, 183-103 bar, 203-109 bar, 222-114 bar; MeOH makeup flow: 10 µL/min; shifted IMS inlet voltage: −6.5 kV, acquisition rate: 10 Hz; chromatogram was processed with a 10 point FFT filter.

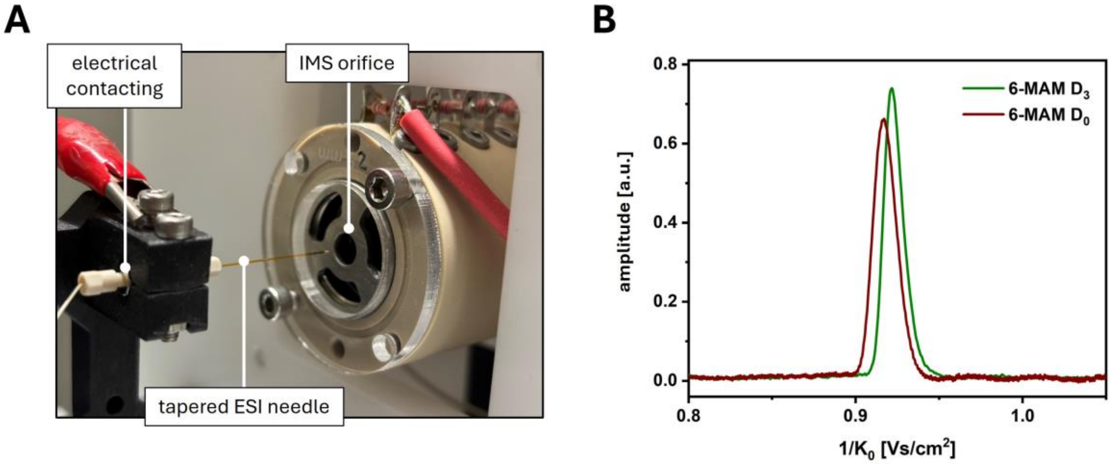

Figure S5 – ESI-IMS measurements of 6-MAM D_3 and 6-MAM D_0. (**A**) Setup for the ESI-IMS measurements. The sample was pumped at a defined flow rate through a tapered capillary in coaxial alignment to the IMS inlet. Electrical contacting of the liquid (0 V) was achieved using a stainless-steel union. (B) overlay of the inversed reduced mobility plot of 6-acetylmorphine-D_3 (6-MAM D_3) and 6-acetylmorphine-D_0 (6-MAM D_0). For each compound solutions with a concentration of 100 µM were prepared in 80:20 v/v MeOH: H_2O. The sample flow rate and ESI voltage were set to 1 µL/min and −5.5 kV, respectively. The calculated K_0 values for 6-MAM D_3 and 6-MAM D_0 were 1.085 cm^2/Vs and 1.090 cm^2/Vs.

Note: Due to legal restrictions, the concentration of commercially available 6-MAM D_0 stock solutions is limited to 100 µg/mL. Consequently, the resulting concentrations of 6-MAM D_0 within mixtures of other compounds were insufficient for SFC-IMS detection using the present setup. Therefore, 6-MAM D_3 was selected as an alternative, given its availability at higher concentrations without legal limitations.

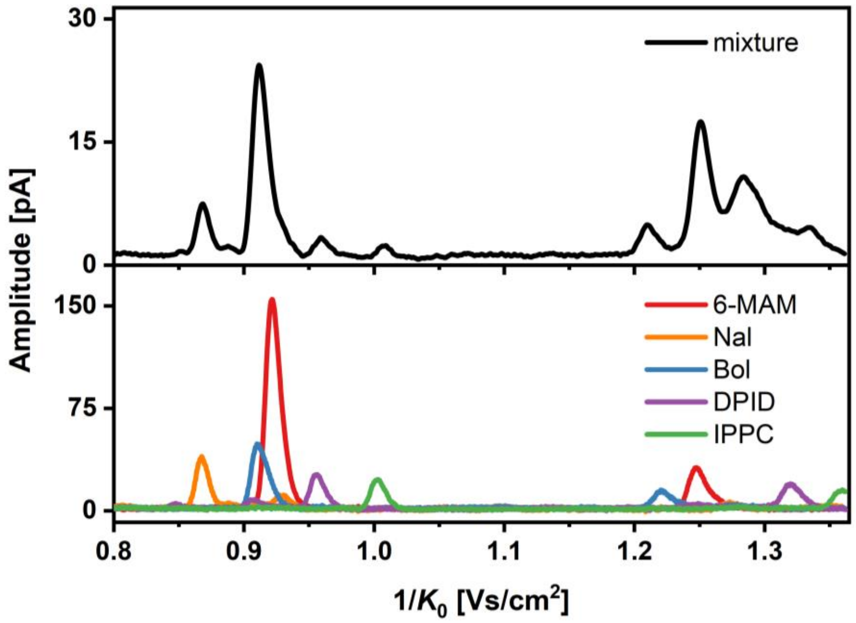

Figure S6 – Inversed reduced mobility plot of the individual mixture components (bottom) and the mixture (top) as measured by direct infusion ESI. Sample: 100 µM of each 6-acetylmorphine-D_3 (6-MAM), naloxone (Nal), boldine (Bol), 2-[3-(4,4-dimethyl-2,6-dioxocyclohexyl)propyl]isoindoline-1,3-dione (DPID) and 2-isopropylphenyl *N*-(4-(ethoxycarbonyl)-phenyl)carbamate (IPPC) in 80:20 v/v MeOH:H_2O, sample flow rate of 1 µL/min, −5.5 kV ESI voltage.

Characteristic drift times and K_0 values of the five mixture components

compound	drift time [ms]*	K_0 [cm^2/Vs]*
6-MAM	**10.58**, 14.32	**1.09**, 0.80
Nal	**9.89**, 10.58	**1.15**, 1.08
Bol	**10.36**, 13.90	**1.10**, 0.82
DPID	10.32, **10.87**, 15.02	1.10, **1.05**, 0.76
IPPC	**11.41**	**1.00**

* bold numbers refer to strong signals (>16 pA)

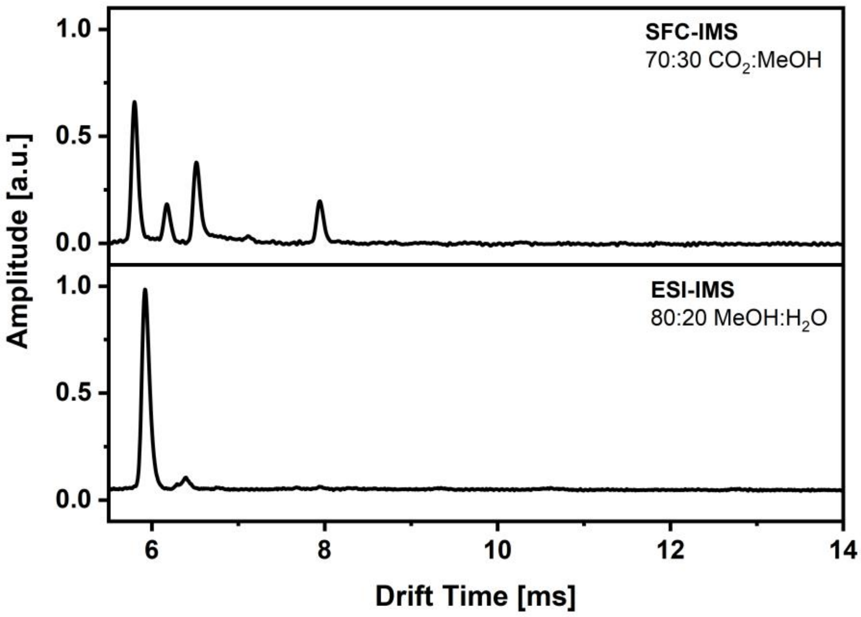

Figure S7 – Drift time spectra of SFC-IMS and ESI-IMS solvent. SFC-IMS: 70:30 v/v CO_2:MeOH; column: 4.0 cm, IA-3, T = 25 °C, pressure drop: $p_{precolumn}$ = 180 bar, $p_{postcolumn}$ = 95 bar; MeOH makeup flow: 10 µL/min; shifted IMS inlet voltage: −6 kV. ESI-IMS: 80:20 v/v MeOH: H_2O, sample flow rate of 1 µL/min, −5.5 kV ESI voltage.
